# Women’s experiences of living with increased inter-recti distance after childbirth: an interview study

**DOI:** 10.1186/s12905-020-01123-1

**Published:** 2020-11-23

**Authors:** Martin Eriksson Crommert, Karolina Petrov Fieril, Catharina Gustavsson

**Affiliations:** 1 University Health Care Research Centre, Faculty of Medicine and Health, Örebro University/Örebro University Hospital, S-building, 701 85 Örebro, Sweden; 2 Centre for Clinical Research, County Council of Värmland, Hus 73, Plan 3, 651 85 Karlstad, Sweden; 3 Centre for Clinical Research Dalarna, Uppsala University, Nissers Väg 3, 791 82 Falun, Sweden; 4 School of Education, Health and Social Studies, Dalarna University, 791 88 Falun, Sweden; 5 Department of Public Health and Caring Sciences, Family Medicine and Preventive Medicine, Uppsala University, Box 564, BMC, 751 22 Uppsala, Sweden

**Keywords:** Diastasis recti, Qualitative content analysis, Postpartum, Women’s health

## Abstract

**Background:**

Although an increased inter-recti distance, also known as diastasis recti, is common after pregnancy, evidence-based knowledge about the condition is relatively limited. In particular, little is known about the consequences as perceived by the women. The objective of the present study was to describe how postpartum women with increased inter-recti distance experience the condition as well as the contacts they have had with healthcare providers regarding their symptoms.

**Methods:**

A purposeful sampling approach was used to recruit 19 participants from an existing study cohort of 144 women. All participants had an inter-recti distance of at least two finger widths and at least one child, with the youngest child between the ages of 1 and 6 years. Individual interviews based on a semi-structured interview guide were performed and subsequently analysed using qualitative content analysis.

**Results:**

Four categories emerged from the interviews: the body’s function and ability has changed; the body does not look like it used to; uncomprehending attitudes and treatment in their surroundings; and trying to acquire an understanding of and strategies to cope with the diastasis. The findings reveal that women with increased inter-recti distance might experience fear of movement and engage in avoidance behaviour. In combination with feelings of physical instability in the midsection of their bodies and body dissatisfaction, many of the women restrict their everyday lives and physical activities.

**Conclusions:**

The findings indicate that increased inter-recti distance is a complex phenomenon that affects the women in a multitude of ways, highlighting the importance of considering the condition for each individual in her own context from a biopsychosocial perspective.

## Background

During pregnancy, the muscle bellies of musculus rectus abdominis are pushed apart and the fascia structure linea alba that is separating the two bellies is stretched out, leading to an increased inter-recti distance (IRD) [1]. Although this happens to almost all women in the third trimester, for around 40% the IRD remains increased 6 months post-partum [1], and there are reports suggesting that the condition might persist in women even after their child-bearing years [2]. Considering the high prevalence of the condition, relatively little research has been conducted in this field.

The increased IRD constitutes a biomechanical change in the structures of the abdominal wall. Findings suggest that the presence of an increased IRD postpartum is negatively related to abdominal muscle function [3, 4]. The motor control and function of the abdominal wall are in turn related to lumbopelvic pain [5]. However, even though an association between increased IRD and lumbopelvic pain has been suggested [4, 6], several findings dispute this relationship [1, 7]. There seems to be no conclusive evidence linking the condition of increased IRD to deteriorated physical function [8]. Thus, we have no clear picture of the condition and its consequences, and the actual symptomatic burden on the affected women has not been established yet. Given the lack of research and the absence of a sound evidence base, there are no clinical guidelines to follow. As a consequence, healthcare professionals are left to let their own beliefs and attitudes guide the treatment, or to make use of information they adapt from online resources with uncertain levels of evidence [9]. Although, recent efforts have been made to reach consensus about the treatment of increased IRD [10], there is still a risk that women with increased IRD receive unequal treatment if they seek help for their condition from healthcare providers.

The biopsychosocial model, originally introduced in the late 1970s, states that an individual with a health condition needs to be understood from a biological, psychological and social perspective [11]. The extent to which each domain contributes to the understanding of a health condition varies between individuals and within individuals over time [12]. We suggest that it is helpful to move beyond strict biomedical or psychological perspectives when exploring a poorly understood condition such as increased IRD.

The aim of this study was to describe how women with increased IRD postpartum experience the condition as well as the contacts they have had with healthcare providers regarding their increased IRD.

## Methods

### Study design

This study had a cross-sectional explorative and descriptive study design and used methods for qualitative content analysis [13, 14] of interview data.

### Participants

Nineteen participants were recruited from a sample of 144 women who had previously participated in data collection for another study. The women in the large sample, who all had an IRD of at least two finger widths and at least one child, with the youngest child being between 1 and 8 years old, had been recruited through advertisements in local newspapers, advertisements at childcare centres and via Facebook. When signing the consent form to participate in the previous study, the women also agreed to be contacted again with regards to the present interview study. A purposeful sampling approach [15] was used in the present study to select participants who represented as much variation as possible concerning the following variables: age, body mass index (BMI), lumbopelvic pain, type of delivery (vaginal/caesarean), twin births (yes/no), number of children, age of youngest/oldest child, and home town. Background characteristics of the 19 participants in the present study are presented in Table 1. Prior to the interviews, verbal and written information about the study was provided to the participants and written consent was obtained. The study was approved by the Regional Ethics Review Board at Uppsala University (No. 2017-316).

**Table 1:** Participant background characteristics (n = 19)

	Mean (SD)	Range
Age	38 (4.8)	30–45
Body mass index	25.22 (5.76)	16.8–36.3
Lumbopelvic pain (0–10)^a^	3.5 (2.5)	0–8
Number of children	3 (1.5)	1–6
Age of youngest child	3 (1.8)	1–6
Age of oldest child	8 (6.4)	1–23
Inter-recti distance (cm)	4.2 (1.6)	2.0–8.8
Twin births (%)^b^	26	
Caesarean (%)^c^	37	

^a^Lumbopelvic pain was rated as the mean pain level over one week on a numeric rating scale of 0 (no pain) to 10 (worst pain imaginable)

^b^Twin births: percentage of women with at least one twin pregnancy

^c^Caesarean: percentage of women with at least one caesarean

### Procedure for data collection

The individual interviews were carried out during the spring of 2019. The participants chose the locations: one interview was conducted at the participant’s healthcare centre, two in the participants’ homes, four at the participants’ workplaces and twelve at research centres in their hometowns. The interviews lasted between 20 and 70 min (35 min on average), were recorded digitally and transcribed verbatim. The first author performed all interviews, following a semi-structured topic guide containing open-ended questions [14] designed to respond to the research questions of the study: (1) How do the women perceive their bodies after pregnancy, particularly in relation to their bellies? (2) How do the women perceive the relation between the rectus diastasis and their physical abilities? (3) If the women had been in contact with healthcare providers regarding their rectus diastasis, what is their experience of the treatment and attitudes they encountered? The interview guide was developed for this study, and is provided as Additional file 1. The women were asked to reflect on their own experiences of having IRD rather than report what they had heard or read about. To obtain data that accurately represented the narratives of the participants and thus increase credibility, the interviewer posed probing and follow-up questions during the interview [16], such as: “Can you tell me more about…?”, “How do you mean?”, “Can you please try and elaborate on…?”. Only the participant and the interviewer were present at the interview sessions.

### Data analysis

The transcribed text files were analysed using qualitative content analysis with an inductive approach [13, 17]. First, to get a sense of the whole, the three authors (who all are physiotherapists) read each interview several times. The first author identified, condensed and coded the meaning units, i.e. specific units of text relating to the research questions. Then, all three authors reviewed and discussed the coding in order to ensure credibility of the presentation of the data. After the coding, all three authors participated in the grouping of codes into higher-order categories and subcategories. NVivo software (NVivo 12, QSR International Pty Ltd, Chadstone, Victoria, Australia) was used to assist the analysis. In order to validate the interpretation and increase trustworthiness of the findings, the authors re-read the text files, discussed the coding, reviewed the preliminary interpretation, and revised the interpretation until consensus was reached [17]. Table 2 provides an example of the analytic process by which meaning units were coded, and subcategories and categories were formed.

**Table 2:** Subcategories and examples of codes and meaning units in the category “The body’s function and ability has changed”

Category	Subcategories	Examples of codes	Examples of meaning units
The body’s function and ability has changed	Disappointed in the body and its ability	Do not trust the body anymore	*…I don’t think I trust my body today as much as I did before the pregnancies*
			*I do not trust the body anymore, before I trusted in it to get me from point A to point B, to go on vacation or to go out for a run… to do stuff*
		Disappointed about loss of a strong body	*You want to be strong, I mean you get irritated on yourself, you get angry with your own body when you feel weak or… fragile…*
			*I do not feel strong anymore… before I felt strong, lively and healthy and I could do stuff*
	Content with the body and what it is capable of, given the circumstances	Content with the body in general	*…the stronger I feel the more content I am with myself. Even if certain things have not changed, like the loose skin does not go away because I get stronger, but I do not care about that, because I feel good in my body*
			*…it does not matter if I am a bit too heavy because of bad eating habits…I feel I am more or less back to normal…*
		The body is good enough – it does not hinder me	*…so the whole body has gone soft, but it is good enough to take care of the kids and to do what I want it to*
			*The body is good enough for me to be able to live the life I want to live right now. I have no pain or other bodily issues so I would say that it is good enough…*
	Negative impact on functioning in daily life	Symptoms from belly during everyday activities	*Oh God yes, I cannot take of my sweater or anything. As soon as I engage my abdominal muscles a bit too much, or bend backwards or something, it pops out ventrally…*
			*…it feels like the insides will be falling out, that is why I cannot carry the laundry basket*
		I have no stable centre	*…as soon as I move I cannot keep it* [the belly] *together. No matter how hard I try, I cannot, it is like it is totally dead*
			*…wobbly is the best word I can think of. It is like… the upper and lower body are not connected anymore*

## Results

The four categories and 13 subcategories are outlined in Table 3. They are described in further detail below and illustrated with quotations from the interviews in italics.

**Table 3:** Overview of the categories and subcategories that emerged in the analysis of the interviews

Categories	Subcategories
The body’s function and ability has changed	Disappointed in the body and its ability
	Content with the body and what it is capable of, given the circumstances
	Negative impact on functioning in daily life
The body does not look like it used to	Ashamed of and sad about the appearance of the belly
	The changed appearance of the body has a negative impact on self-image and self-esteem
Uncomprehending attitudes and treatment in their surroundings	Lack of understanding from the surrounding community
	The healthcare system does not know and does not care
	My partner does not understand why I cannot be the same anymore
Trying to acquire an understanding of and strategies to cope with the diastasis	Have searched for information on their own to learn about the diastasis
	Have learnt about the diastasis from own bodily experiences
	Struggle with acceptance through conciliation with the body’s abilities and appearance
	Avoid activities of daily life and physical exercise
	Have not thought much about the diastasis

### The body’s function and ability has changed

Some of the interviewed women were satisfied with the way that their bodies were functioning after their pregnancies. They acknowledged that the body paid a price for being pregnant, but that it was worth it. Some even expressed that they were more content with the way their bodies functioned after they gave birth.

It’s hard to say, because I think it is about a maturation process, so, I don’t know. Right now, where I am today, I’m more content with my body than before. Participant 6.

Most of the women however expressed a sense of disappointment and felt that they no longer could rely on their bodies the way they used to because they did not perceive their bodies as strong anymore. For some, this even came to the point where they felt ashamed and sad because they could not get their bodies to function like before, and they grieved their loss.

Yes, it affects me, I become sad, and…yeah sad on a deeper level. That I can feel depressed today about something that I once built, to be strong, has been destroyed and that it happened so quickly. Participant 4.

Many women also reported that the increased IRD affected their daily lives and that they felt discomfort or painful sensations mostly from the belly, but also from the lumbopelvic region. Some used the words ‘muscle cramp’ to describe the sensation, whereas others thought it was a more diffuse sensation that was hard to describe. They expressed sadness because it hindered them from many everyday activities, such as lifting heavier loads, e.g. laundry baskets, or playing around with their children in such a way that they could get pushed in their bellies.

The children still climb on me from time to time, that doesn’t work. ‘No, you can’t sit there, you can’t lie there because it hurts’. It’s not heavy, but it’s like, I have nothing to resist with. Participant 6.

Furthermore, many of the women, regardless of whether or not they experienced discomfort or pain, described a sensation of heaviness in their pelvic region or of their belly falling out, already at relatively low loads.

Yes, oh God yes, it’s enough for me to take off my sweater, as soon as I engage my abdominal muscles a bit too much, or bend backwards or something, it pops out ventrally. Participant 19.

Most of the women described that they had difficulties recruiting their abdominal muscles properly; they expressed that they did not ‘find’ them, leaving them with a sense of instability in the mid-section of the body. Among other things, the women described it like they lacked a centre, that they could not keep their belly together, that their mid-section was ‘dead’, and that they lacked support in the middle. Several women described that they constantly needed to think about activating their abdominal muscles in order to get at least some sense of stability since they experienced no automatic activation anymore. During daily activities and exercise, the body felt wobbly. During e.g. physical exercises, they preferred to use seated or lying positions due to the perceived lack of muscular control/stability in standing positions. Some women felt that an elastic belt gave them a positive sense of support, others did not.

But it doesn’t really feel like it’s possible to tighten them, and they don’t respond the same way as before, you have to focus more on that part to engage the body. Participant 2.

And then, I can’t do things, in certain classes, even during yoga, I can’t. There is a lot of stuff I can’t do because it takes its toll on the belly, even if I have one of those abdominal supports or training corsets, it won’t stay together. Participant 17.

Especially in the evenings, after a meal or after physical exercise many women experienced that their belly got taut and swollen. Some of the women described that they associated the increased IRD with poorly functioning bowels.

I experience partly that I get a more negative approach to food because of my diastasis. In the morning, the belly is flatter, and it is easier to recognise my body. But with more and more meals in the belly, it gets bigger in an unnatural way. Sometimes I get tempted to hold back on food because it makes such a huge difference. It is nothing that I’m proud of, but it is a direct solution to the problem, at least for that same day. Participant 3.

### The body does not look like it used to

While the vast majority of the interviewed women acknowledged that there were some changes to the shape and appearance of their belly, which they associated with the increased IRD, a few did not render it any importance. Most of the women however expressed feeling ashamed of their belly and many described a constant awareness of their belly bulging outwards, looking pregnant. Others tried to repress their belly from their conscience.

I’m trying to erase the image of my belly, I don’t want it. So, it’s my lower body and my upper body from the breasts and up. I don’t want to see the belly, when I see it I become sad, so I’m trying to avoid that as much as I can. Participant 17.

Some of the women described a constant and ongoing effort of relating to their body and inducing a self-acceptance of their new appearance. They experienced a loss of the ability to feel proud of their body, which lowered their self-esteem. Most of the women in some form expressed a reluctance to show their belly to others, mainly describing that they had stopped wearing bikinis to the beach or swimming pool, but also that they no longer changed clothes in shared dressing rooms or that they hid their belly in large loose-fitting clothes. But the feelings of shame and the negative body-image were also described by several women as having a large negative impact on their relationship with their partner. Several women did not consider themselves attractive anymore and described a loss of desire for sex.

Well, I’m really uncomfortable with him now, really, and he knows that, too. I don’t like closeness like that anymore…I think it is hard because I feel like a big lump. Yeah, that’s how it is. So it affects…like a big part…really. Participant 16.

### Uncomprehending attitudes and treatment in their surroundings

A vast majority of the women had sought help regarding their increased IRD from the healthcare system. While a few women experienced that they had been listened to and treated respectfully, most of the women reported that they had been met with a demeaning attitude and that their problems had been trivialised. They experienced an ignorant attitude regarding the condition among healthcare professionals who generally had no idea about how to treat their condition or where to refer them. Many of the women expressed that they could not be bothered to contact the healthcare system again, because they did not think it would be worth the effort.

You felt rather stupid, like you had wasted their time and that they thought you had sought help because…, I don’t know…, it felt like, – I felt stupid, like a waste of my time and theirs, and a feeling of – Ok, so this is how it is going to be… You felt rather abject…Participant 6.

A common experience was that increased IRD as a condition is starting to get a foothold in the media and the public domain. However, a majority of the women described episodes where they had been met with ignorance and lack of understanding from their surroundings. Several of the women described that they felt stigmatised in different situations, e.g. when they participated in gym-classes and had difficulties performing all exercises. Another aspect that was commonly reported was the feeling of having to explain themselves to colleagues at work or to new acquaintances with regards to the appearance of their belly, for example by saying ‘*No, I am not pregnant*’, or that they constantly needed to ask for help carrying even relatively light loads.

You become sad about having to explain it to every person you meet who presumes that you function in a certain way, you have to explain. Participant 4.

While many of the women expressed negative concerns with their appearance, most of them described acceptance from their partners. However, some felt that they could not live up to their partner’s expectations in everyday life, e.g. managing household chores, or in their intimate relations. One woman explicitly said that her husband had been appalled by the appearance of her belly after she gave birth to twins. However, mostly the partners had difficulties understanding that the women themselves did not have the same desire for closeness and sex as before because of their own issues with their bodily appearance and functional capacity.

So, when I’m with my partner, with him I have to explain why I don’t function as before, that it’s not because, that I’m saying no to a sex-life, is not because of him, but because of me. Participant 4.

### Trying to acquire an understanding of and strategies to cope with the diastasis

Almost all of the interviewed women expressed that they had searched for information on their own to learn about increased IRD and how to manage it, but that they had had difficulties in finding information. A majority felt that they did not know enough. They had learned what they knew from books, magazines and online searches, i.e. blogs and postings from personal trainers and media personalities who write about women’s health issues. They found it hard to assess whether the information was credible and based on something other than just opinions and beliefs. This made it difficult to know how much and what type of physical exercise they could do, which for many women resulted in not exercising at all.

I don’t know if there’s like research behind it, or if it is just guessing or what it is, I’m not sure. Participant 12.

Most of the women, however, had gained information from a popular exercise app for new mothers on a specific type of low load exercises to be performed several times a day. The exercises were perceived as very boring, and most women did not perform them regularly even though they admitted that it felt better when they did.

Due to the lack of knowledge, many women tried to act according to what felt right in their bodies. Some of them exercised more than they did before their pregnancies since they felt that it was necessary in order to maintain their functional level. However, many of the women also described that they avoided doing certain activities altogether because they felt discomfort or pain performing them or because they sensed that they did not have the strength or proper trunk muscle control to perform them anymore. They also described how the mere anticipation of discomfort and/or pain triggered avoidance of activities because of fear that they might worsen their condition, so that they would get symptoms in the future, even if they did not have any complaints to date. This effectively excluded them from some of the social contexts they had been part of previously.

It is related to the fact that you can’t count on, count on the body the same way, and because you are afraid and anxious you hesitate before you do certain things, which means that you are even more afraid and anxious when you actually have to do those things. Participant 4.

And the greatest fear is to make it even worse, that you will make it go even deeper…even wider…, so that you will get an even bulgier belly and so…it can sometimes be tempting to bunk off exercising. Participant 3.

For most women, strategies to cope with the increased IRD involved making adjustments to everyday-life activities, such as adopting a slower walking pace, changing the type of physical exercise they perform or playing with their children more calmly. Some women also described that they had started to avoid certain activities and exercise because of advice given to them in their exercise app, which explicitly stated that they needed to master certain exercises before they could move on to more complex and heavy activities.

Many of the women had come to terms with the changed appearance of their body and the experience of limitations in activities of daily life associated with the increased IRD. Yet, the process of acceptance was not easy. Most women experienced, or had experienced, a process of conciliation where they gradually and reluctantly accepted a new life with compromises.

Now it’s been more than a year and a half so now I have conciliated with how things are, but still, it doesn’t feel good. Participant 2.

Whereas most of the women described several adjustments to and/or avoidance of certain activities to cope with the perceived consequences of the increased IRD, there were a few women who reported that they had not thought much about their condition at all. These women felt no fear or worry that the condition would progress and had not sought any help regarding the increased IRD because it had not bothered them.

I haven’t cared about it, because it hasn’t bothered me. It has been like ‘Oh shit, look I can fit two fingers in. Oh well, I couldn’t do that before’. Participant 8.

## Discussion

In this study, women reflected upon their experiences of having increased IRD. Increased IRD emerges from these interviews as a complex condition that affects the women in a variety of ways, and the severity of the perceived consequences of the condition on their lives varied greatly.

A very prevalent physical experience among the participating women was a changed sensation in and a changed relation to the mid-section of their body. Most women described weakness, lack of connection or lack of trunk control and a sense of instability or wobbliness. It is possible that the biomechanical change in the abdominal wall is at least partially responsible for these experiences. The muscles of the lateral abdominal wall are extensively linked to the control of the stability of the lumbar spine and pelvis [18, 19]. An increased width of the linea alba would affect the length of the muscles in the lateral abdominal wall, especially musculus transversus abdominis, which has a mainly horizontal fibre orientation [20]. This has the potential to affect the length-tension relationship and make the muscles inefficient [21]. Furthermore, a larger IRD would result in a larger portion of the abdominal wall having no contractile ability, further affecting the stabilising effect of abdominal muscle contraction. The women’s experiences of pain or discomfort were related to the abdomen to a larger extent than to the lumbopelvic region, while the latter has received almost exclusive attention in the literature [1, 7]. This problematizes the hypothesised link between altered muscular function and lumbopelvic pain in this group of women and should be addressed in future studies.

For many of the participating women, it was hard to accept the altered body constitution. However, concern and dissatisfaction with body shape and weight is common among postpartum women [22, 23]. For this reason, this finding cannot specifically be accredited to the increased IRD. However, considering the high prevalence of increased IRD postpartum, it is likely that the study populations in previous studies also have included several women with this condition. Furthermore, a prospective study has shown that the peak of dissatisfaction among postpartum women lies at around 6 months postpartum [24]. The women in the present study were between 1 and 6 years postpartum, which perhaps indicates that the dissatisfaction in women with increased IRD is more persistent than previously shown. Perhaps the sense that something is ‘wrong’, expressed as not finding the abdominal muscles or not being able to recruit the abdominal muscles properly, could lead to even more negative attitudes towards one’s own body. The negative beliefs about their belly led some of the women to avoid sexual contact with their partner and they expressed a decreased sexual desire. This finding is also commonly reported in the literature in postpartum women (cf [25].). However, the reasons given previously have been e.g. lack of time, tiredness and breastfeeding [26]. In the present study however, the women blamed the increased IRD for changing their belly’s physical appearance, which affected their libido negatively. Thus, although decreased sexual desire among postpartum women has been reported previously, the underlying reason for this decrease is different in our study.

The women in this study described that they did not have enough knowledge about their increased IRD. Such a desire to know more about one’s condition is sometimes labelled uncertainty reduction, a need to reduce the subjective level of uncertainty [27]. However, the women in the present study expressed difficulties in finding information to help them understand how to cope with their condition. In the absence of answers from healthcare providers, they turned to other sources of information, such as social media, blogs, magazines or books. Somewhat paradoxically, the women perceived these sources of information—i.e. the person behind the blog for example – as trustworthy, while most of them at the same time were aware of and often frustrated about the lack of evidence behind the information. A potential risk of leaving women to search for information themselves is that there might be an increased tendency for confirmation bias, i.e. a risk that they give preference to information that strengthens their own views and perspective and disregard information that questions them [28]. Avoidance of information that would cause a mental dissonance is a well-known issue in health communication [29], thus not unique to the present context. However, it could constitute a particular problem in the present situation due to the lack of comprehensive evidence-based information provided by the healthcare authorities.

As a potential consequence of the lack of information based on scientific evidence, many of the women had received an ignorant and diminishing treatment from healthcare providers. The women’s response was to give up. Even though their problems did not always decrease over time, they simply could not be bothered to seek help. In a recent study, we showed that midwives and physiotherapists also are frustrated about the scarce evidence base for the management of increased IRD [9]. But it is unfortunate if healthcare professionals adopt attitudes that can be perceived as negative or patronising because of their own uncertainty. However, the women experienced uncomprehending attitudes not only from healthcare providers but also from the community and even from their own partners. They expressed a need to frequently explain themselves, not only with regards to their appearance but also their decreased physical ability. This might be a nuisance during the first year postpartum but can easily be perceived as becoming a significant problem when it persists over a longer period of time, and this had in many cases led the women to avoid participation in social and physical activities.

An avoidance of movements or activities that are unpleasant, or believed to be unpleasant or painful if they were to be performed, is referred to as fear-avoidance behaviour. According to the fear-avoidance model, such behaviour is one contributing factor to the transition from acute to chronic pain [30]. In the present study, many women expressed concerns that they might worsen their condition or get future pain problems if they engaged in for example certain activities or the wrong exercises. The fear of worsening their condition was present even though the exercises as such did not provoke pain or discomfort. In order to target possibly unfavourable fear-avoidance behaviour and to provide self-management advice to women with increased IRD, there is a need of further studies that investigate the effects of various physical activities and exercise interventions in women with increased IRD.

We argue that we can aid the understanding of the complexity of the phenomenon and the plethora of perceived consequences of increased IRD by considering the various domains of the biopsychosocial model [11]. Although the structural alteration of the abdominal wall can be considered in isolation as a strict biological issue, this point of view seems severely limited. The impact on the biological domain, e.g. decreased muscle function and the experience of instability, appears to be closely linked to fear of movement and/or fear of pain in the psychological domain. Associated with the previous factors is the avoidance behaviour that leads to withdrawal from participation in activities of daily life in the social domain. Thus, to be the most effective, treatment should preferably address all these domains.

The present study recruited participants from a sample collected for another study. This can be seen as a limitation since it narrows the recruitment base. However, owing to wide inclusion criteria in the larger sample, it was possible to include participants in the present study who displayed a large variation in both the width of the increased IRD as such and in other background characteristics. For example, the participants’ accounts of their encounter with healthcare providers reflect experiences from cities of varying sizes and several different regions in the middle of Sweden, which all have their own healthcare organisation. This increases transferability to other Swedish regions and contexts and contributes to a high level of trustworthiness. There was also variation in the experiences associated with the increased IRD presented during the interviews, suggesting that the recruitment strategy did not severely limit or bias the outcome of the study. To ensure credibility, i.e. obtain results that accurately represent the narratives of the participants, the interviewer regularly posed follow-up questions, restated and summarised information during the interviews to confirm the accuracy of the material, known as respondent validation [16]. In addition, during the analysis, the authors frequently went back to the transcripts resulting in confirmation of the analysis or in the relabelling or regrouping of codes and so on. Trustworthiness was further strengthened since all authors conducted the analysis together. In addition, since all of the women had participated in the previous study, the interviewer had met them all before the interviews. Thus, the interviewer had been able to build rapport in advance, which probably increased the sense of security in the interview situation and likely increased the richness of the material. Qualitative research designs are imperative in the understanding of various conditions. Future studies that draw on our work are warranted. Among others, studies by methodology of phenomenology looking at the issues of lived experience of having increased IRD, could potentially elucidate further knowledge that may inform clinical care. But also studies using quantitative methods are needed, investigating, e.g. risk factors and inter-relationships to mention but a few aspects.

## Conclusions

The findings in this study reveal that women with increased IRD experience a lack of evidence-based information about the condition, in part contributing to fear of movement and avoidance behaviour. A feeling of physical instability in the midsection of their bodies requires the women to make changes to and restrict their everyday life and physical activities. Added to this, other findings such as body dissatisfaction are presented. Altogether, the findings indicate that increased IRD is a complex phenomenon that affects the women in a multitude of ways, highlighting the importance of considering the condition for each individual in her own context from a biopsychosocial perspective.

## Supplementary information

**Additional file 1**. Interview guide.

## Data Availability

The datasets generated and analysed during the current study are not publicly available as they consist of quotes by the interview subjects that might contain information, which could reveal the identity of individuals. But datasets are available from the corresponding author upon reasonable request.

## Abbreviation

IRD: Inter recti distance
