# Supplementary material for: Women’s experiences of living with increased inter-recti distance after childbirth: an interview study
Source: BMC Womens Health. 2020 Nov 23;20:260. doi: 10.1186/s12905-020-01123-1 (PMC7684710; doi:10.1186/s12905-020-01123-1)
Supplement: Supplementary file 1 — Additional file 1. Interview guide. [file 12905_2020_1123_MOESM1_ESM.docx]

Interview guide

As a rule, the interviewer only asked the first level questions. The second and third level questions were noted in the guide as an aid to get the interviewed women to elaborate some more on the topic when the response to the first level question was very short.

- First, can you briefly tell me about yourself: how old you are, how many children you have and how old they are?
- Are you currently working?
  - What kind of job? Physically demanding or not?
  - Are you experiencing any differences in your work compared to before your pregnancy(ies)?
  - If not working, why is that?
- What do you do in your spare time?
  - What kind of activities? Physically demanding or not?
  - Are you experiencing any differences in your spare time activities compared to before your pregnancy(ies)?
- What do you know about diastasis as a phenomenon?
  - Where did you get the information?
  - Do you feel that you know enough?
- Are you concerned about your diastasis?
- Do you have any pain condition (e.g. pelvic- or low back pain) that started with (any of) your pregnancy(ies)?
  - What do you think is the cause of your pain?
- What do you think of your body after your pregnancy(ies)?
  - Happy or not?
    - Has that changed with the pregnancy(ies)?
    - Is that affected by your diastasis?
  - Do you trust your body?
    - If not, what part of it do you not trust?
  - How do you feel your body is functioning (e.g. lower back, pelvic floor)?
    - Is there a difference compared to before your pregnancy(ies)?
- Have you been in contact with any healthcare professionals regarding your diastasis?
  - If so, what profession?
  - How was your experience from that/those occasions?
  - What do you think about the advice/treatment you received from those health professionals?
